# Commentary: Cluster failure: Why fMRI inferences for spatial extent have inflated false-positive rates

**DOI:** 10.3389/fnhum.2017.00345

**Published:** 2017-06-28

**Authors:** Karsten Mueller, Jöran Lepsien, Harald E. Möller, Gabriele Lohmann

**Affiliations:** ^1^Nuclear Magnetic Resonance Unit, Max Planck Institute for Human Cognitive and Brain SciencesLeipzig, Germany; ^2^Department of Biomedical Magnetic Resonance, University Hospital TuebingenTuebingen, Germany; ^3^Magnetic Resonance Center, Max Planck Institute for Biological Cybernetics, TuebingenTuebingen, Germany

**Keywords:** fMRI, functional magnetic resonance imaging, false positive results, false positive error, random field theory, family-wise error rate

In a recent manuscript, Eklund et al. (2016) reported inflated false positive rates in functional MRI (fMRI) using several common software packages. Here we would like to draw attention to an important aspect that was not addressed in this publication. Specifically, we would like to note that statistical inferences obtained using the random field theory depend heavily on a preprocessing parameter not discussed by Eklund et al. (2016), namely the spatial resolution to which the data sets are resampled and interpolated during pre-processsing. This resampling is needed to align the data to a common anatomical template that is essential to perform group analyses (also often called *normalization*). Eklund et al. (2016) used the default setting of 2×2×2 mm^3^. In response to Eklund's paper, Flandin and Friston (2016) used a different setting of this parameter, namely 3×3×3 mm^3^. Together with a more stringent initial cluster-forming threshold, they did not observe inflated false positive rates. However, the 2×2×2 mm^2^ setting is the default in two major software packages (SPM, FSL), and in previous work, Friston and colleagues Hopfinger et al. (2000) stated that resampling to 2×2×2 mm^3^ renders the analysis “more sensitive.” In other words, at present it is unclear what a valid setting for this parameter should be. Therefore, we think that it is extremely relevant to assess its influence on statistical inference.

For this purpose, we analyzed a group of 47 resting-state fMRI data sets with a spatial resolution of 3×3×4 mm^3^ and 300 volumes used in a preceding study Mueller et al. (2016). Using a strategy analogous to Eklund et al. (2016) we imposed various fake designs including block- and event-related types. We tested the following resampling parameters: 3×3×4, 3×3×3, 2×2×2, and 1×1×1 mm^3^. Using SPM12 with family-wise error (FWE) correction based on the random field theory, we first evaluated each data set separately. We found that with higher resampling resolutions, the FWE-corrected *p*-values decrease systematically so that more and more false positives occur. Figure 1A shows a typical result. We obtained a systematic effect in all of the 47 data sets (Figure 1B). We also observed a systematic effect of image upscaling onto smoothness estimation (Figure 1C). Furthermore, we performed a group-level inference in which all 47 data sets were pooled. Again, we observed that the FWE-corrected *p*-values decreased systematically with higher resampling resolutions.

**Figure 1:**
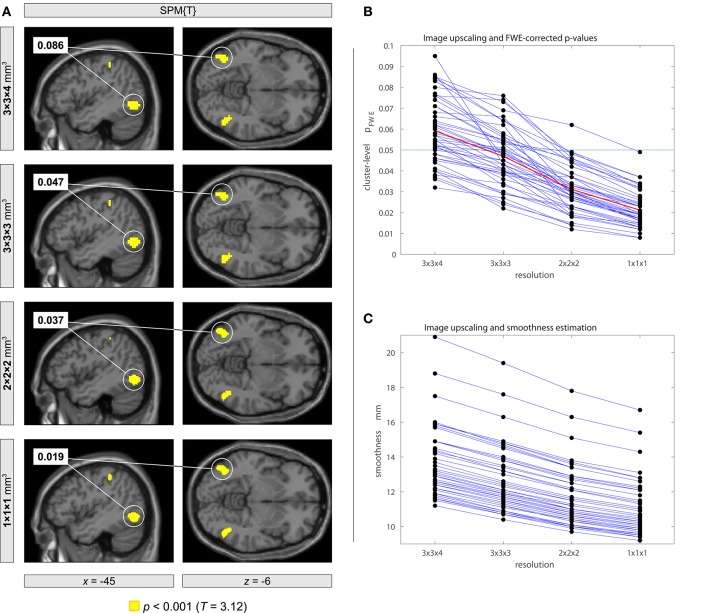
Impact of using different image resolutions onto family-wise error (FWE) corrected *p*-values with the analysis of resting-state fMRI data using a fake on-off block design. The statistical model included an experimental condition with 18 blocks with a block length of 20 s using an onset every 40 s. The same statistical analysis was performed with different image resolutions obtained within the normalization step in SPM12 followed by a subsequent spatial smoothing of 8-mm full width at half maximum. **(A)** Orthogonal brain sections of an individual subject showing a cluster of brain activity that becomes significant when using image upscaling. **(B)** Systematic decrease of FWE corrected *p*-values with increasing image resolution for each participant. The plot shows *p*-values for a randomly selected cluster with *p* < 0.1 for each participant. On average, *p*-values became smaller than the half size of the original value (see line plotted in red color). **(C)** Systematic decrease of the estimated smoothness when using image upscaling.

In other words, it appears that there is a *systematic* dependence of the false positive rate on the resampling parameter with smaller voxel sizes leading to smaller FWE-corrected *p*-values and hence more false positives. While some dependence on preprocessing parameters may be inevitable, a systematic dependence of this type is clearly worrisome, because researchers may be tempted to interpolate their data until the desired statistical significance level is reached. Statistical inference should certainly not depend in such a systematic way on a preprocessing parameter that can be set *ad libitum*. Clearly, this issue requires further in-depth analysis.

## Author contributions

KM communicated carried out the data analyses, and wrote the first draft of the letter. All authors assisted with the conceptual approach and contributed to the writing.

### Conflict of interest statement

The authors declare that the research was conducted in the absence of any commercial or financial relationships that could be construed as a potential conflict of interest.
